# The Pain Intensity/Quality and Pain Site Association with Muscle Activity and Muscle Activity Distribution in Patients with Chronic Low Back Pain: Using a Generalized Linear Mixed Model Analysis

**DOI:** 10.1155/2022/5751204

**Published:** 2022-05-25

**Authors:** Hayato Shigetoh, Yuki Nishi, Michihiro Osumi, Shu Morioka

**Affiliations:** ^1^Department of Physical Therapy, Faculty of Health Science, Kyoto Tachibana University, Kyoto, Japan; ^2^Neurorehabilitation Research Center, Kio University, Nara, Japan; ^3^Department of Rehabilitation Medicine, Nishiyamato Rehabilitation Hospital, Nara, Japan; ^4^Department of Neurorehabilitation, Graduate School of Health Sciences, Kio University, Nara, Japan

## Abstract

**Background:**

Pain can alter muscle activity, although it is not clear how pain intensity and site location affect muscle activity. This study aimed to reveal the complex associations among the pain site, pain intensity/quality, muscle activity, and muscle activity distribution.

**Methods:**

Electromyographic signals were recorded from above a bilateral lumbar erector spinae muscle with a four-channel electrode in 23 patients with chronic low back pain while they performed a lumbar bending and returning task. We calculated the average value of muscle activity during the extension phase (agonist activity) and the centroid of muscle activity, as well as the distance between the centroid of muscle activity and pain site. We also assessed the pain site and pain intensity/quality by the interview and questionnaire method. A generalized linear mixed model analysis was performed to determine the relationships among pain intensity/quality, pain site, and muscle activity.

**Results:**

The results showed that muscle activity during the extension phase was significantly negatively associated with neuropathic pain and “pain caused by light touch.” In addition, the distance between the centroid of muscle activity and pain site during the extension phase was significantly positively associated with intermittent pain, “throbbing pain,” “splitting pain,” “punishing-cruel,” and “pain caused by light touch.”

**Conclusions:**

Our findings suggest the existence of a motor adaptation that suppresses muscle activity near the painful area as the pain intensity increases. Furthermore, the present study indicates that the presence or absence of this motor adaptation depended on the pain quality.

## 1. Introduction

Interventions focused on movement and muscle activity are implemented for rehabilitation of many patients experiencing musculoskeletal pain because pain and muscle activity are interconnected [1]. A pain adaptation model has been used to explain the relationship between pain and muscle activity [2]. This theory suggests that the agonist muscle activity is decreased in order to protect against pain and the antagonist muscle activity is increased. In recent years, in addition to the pain adaptation model, various pain-related factors, such as psychological factors and body perception disturbances, affect muscle activities through the nervous system [3]. It has been reported that patients with low back pain (LBP) have altered erector spinae muscle activity during walking, trunk flexion, and upper extremity raising movements [4]. Focusing on trunk extension, the erector spinae muscle activity was reportedly reduced in patients with LBP when extending from trunk flexion [5]. Other studies have reported that the muscle activity of the erector spinae muscle increases when extending from trunk flexion after thermal nociceptive stimulation [6]. Although previous results seem controversial, it may be stated that pain can alter muscle activity during lumbar extension from a trunk flexion position.

In recent years, the relationship between pain and muscle activity distribution has received a lot of attention. Much of this research has been undertaken through experimental pain. Several studies have reported that experimental pain altered the muscle activity distribution at various sites, including the shoulder and knee [7, 8]. Regarding clinical pain, the previous study reported the lack of variability in the distribution of muscle activity in patients with chronic low back pain (CLBP) [9]. Other studies on LBP have also reported similar findings [10]. Therefore, both experimental and clinical pain can alter the muscle activity distribution. In addition, greater pain intensity is associated with pain quality, such as neuropathic pain [11]. However, it is not fully understood how the muscle activity distribution is affected by pain intensity or quality. It is also not clear whether the distance from the centroid of muscle activity to the pain site correlates with the pain intensity and quality.

Identifying these relationships may lead to a detailed assessment of muscle activity characteristics and, furthermore, to tailor-made interventions. The aim of the present study was to reveal the comprehensive associations among the pain site, pain intensity/quality, muscle activity, and muscle activity distribution. We hypothesized that greater pain intensity inhibited muscle activity near the pain site in order to not enhance pain. In addition, if pain intensity could be related to pain quality, we also expected a relationship between pain quality and muscle activity.

## 2. Materials and Methods

### 2.1. Patients

We recruited 23 patients with CLBP (10 males and 13 females) aged 49–80 years (71.7 ± 9.3 years, mean ± standard deviation (SD)) who had received physical therapy and were enrolled when they suffered from LBP lasting for >3 months [12]. We have also included patients whose LBP region was defined as the one bounded by the lowest palpable ribs superior to each other and the gluteal folds inferior to each other [13]. It was scored ≥1 on a numerical rating scale (NRS) for pain intensity. Also, we categorized patients' LBP into nonspecific LBP (*n* = 6) and specific LBP (*n* = 17). The specific LBPs included spinal stenosis (*n* = 3) and lumbar osteoarthritis (*n* = 14). They were excluded if they had some central nervous system disease, dementia, LBPs that appeared over a period of 3 months, and difficulty understanding questionnaires and tasks. Patients who reported their LBP getting worse over time were also excluded. The study's protocol is conformed to the Declaration of Helsinki. Before the study started, participants provided written informed consent. This study was approved by the Ethics Committee of Kio University Health Sciences Graduate School (approval no. H30-06).

### 2.2. Evaluation of Patient Characteristics Using the Questionnaire

For each patient, the following characteristics were evaluated: demographic data (age, gender), pain duration, pain site, and pain intensity/quality (by the NRS for pain and the Short-Form McGill Pain Questionnaire-2 (SFMPQ-2)).

Each patient was asked to indicate the most painful site with their finger. Next, the distance between the top of the electromyography (EMG) sheet and the painful site was measured, with the top of the attached EMG sheet as the starting position (0 cm).

For pain intensity assessment, we used the NRS for pain (0: no pain, 10: worst pain imaginable). We used the following metrics for pain quality evaluation: items that rate 22 pain quality levels and each quality's intensity on the 11-point NRS [14]. We calculate the total score of each item from the sum of the 22 items. A higher score indicates worse pain. It is shown in the SFMPQ-2 that the system has four subclasses: one affective and three sensory (continuous pain, intermittent pain, and neuropathic pain) subclasses. Affective includes “tiring-exhausting,” “sickening,” “fearful,” and “punishing-cruel.” Continuous pain includes “throbbing pain,” “cramping pain,” “gnawing pain,” “aching pain,” “heavy pain,” and “tenderness.” Intermittent pain includes “shooting pain,” “stabbing pain,” “sharp pain,” “splitting pain,” “electric-shock pain,” and “piercing.” Neuropathic pain includes “hot-burning pain,” “cold-freezing pain,” “pain caused by light touch,” “itching,” “tingling or pins and needles,” and “numbness.”

### 2.3. Lumbar Bending and Returning Task

**Figure 1:**
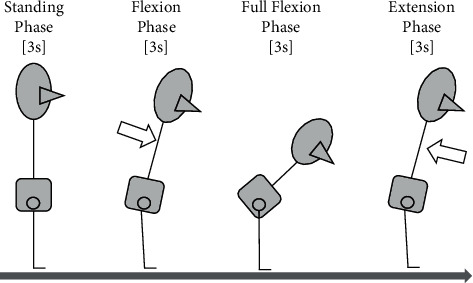
The lumbar bending and returning task.

We asked every one of 23 patients to perform a lumbar bending and returning task, respectively, and each patient was recorded with EMG. For this task, the phases were classified into standing phase, flexion phase, full flexion phase, and extension phase, with each phase lasting 3 seconds (Figure 1) [15].

The patient started the tasks using a stand with no movement (standing phase) with his or her feet at hip width. When the first auditory signal is received, the patient bends forward with slow and controlled motion (flexion phase) to achieve the maximal trunk flexion. Then, the patient was requested to keep the full flexion position (full flexion phase) up to a third auditory signal level. The patient returned to the upright posture after a third auditory signal for 3 seconds. There was a 3-second interval between each auditory signal. We repeated the task for three trials for each patient after completing the reference trial at least once. All patients did not experience LBP appearance during task execution.

### 2.4. Recording and Analysis of Muscle Activities

**Figure 2:**
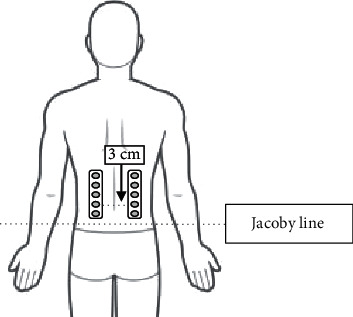
Approximating the EMG lattice location. A 3 cm side-by-side EMG electrode grid was placed on the lumbar spinous process.

An EMG surface signal was detected through an electrode lattice of sheet type (Unique Medical Co., Brooklyn, NY, USA). We used a grid consisting of five electrodes (four channels, with a 25 mm interelectrode distance in both directions). We cleaned the glabrous skin of a patient on the erector spinae by using alcohol. We taped two grids of sheet-type electrodes coated with an electroconducting gel at a given location. An electrode was placed 3 cm away from the midpoint of the lumbar spinous process and superimposed upward along the erector spinae from the level of the Jacoby line (Figure 2). In the left-side radial styloid process, a reference electrode was placed. Then, the recorded EMG signals (sampling rate: 1000 Hz) were analyzed after band-pass filtering (10 and 400 Hz), respectively. At each phase of motion, the root mean square (RMS) EMG values were computed in both the reference trial and the experiment trial. After that, the normalized RMS values of the EMG in each phase were, respectively, obtained by dividing the mean RMS value of the experimental trials by the mean RMS value obtained in the extension phase of the reference trial [6]. For analysis, the RMS value was excluded if the noise could not be removed.

To characterize the muscle activity distribution, the coordinates of the RMS (*y*-axis coordinates for the cranial-caudal direction) centroid were extracted from EMG signals [9]. In each phase, we extracted the centroid. The mean of the right and left centroids during the extension phase of the three trials was used as the centroid of the muscle activity in the statistical analysis. These EMG data were analyzed with custom-written MATLAB code (v.2019b, MathWorks, Natick, MA, USA).

### 2.5. Statistical Analysis

In the extension phase, we extracted the muscle activity and the centroid of muscle activity. The distance between the centroid of the muscle activity and pain site (DISTANCE) was also calculated. For the statistical analysis, the variables used were pain intensity, muscle activity in the extension phase, and DISTANCE in the extension phase.

We performed a generalized linear mixed model (GLMM) analysis to assess the association between pain site, pain intensity, and muscle activity. Considering the sample size of this study, GLMM analysis adopted a Bayesian method that enables reasonable estimation even when the sample size is small. We took a Bayesian approach and explored the Markov chain Monte Carlo (MCMC) fitting of GLMM [16], including the following as fixed effects: pain quality and pain intensity. Participants were included as a random effect. MCMC is a method of generating a sample having a distribution characteristic matching the posterior distribution by the Markov chain using the Bayesian method and using it to calculate an estimated value of the objective variable. We used uninformative prior distribution as our prior distribution, and all iterations were set to 16,000, burn-in samples were set to 13,000, and the number of chains was set to 4. To check the modeling assumption, we used the value of Rhat. A Rhat less than 1.1 for all parameters indicated a good estimation for the model [16]. In addition, all credible intervals (CI) were given with a 95% CI.

We created five models with the dependent variables being muscle activity during extension and “the distance between the centroid of muscle activity and pain site,” respectively: (1) first model: the independent variables were set to SFMPQ-2 subclass (continuous pain, intermittent pain, neuropathic pain, and affective); (2) second model: the independent variable was set to the item of continuous pain (“throbbing pain,” “cramping pain,” “gnawing pain,” “aching pain,” “heavy pain,” and “tender”); (3) third model: the independent variable was set to the item of intermittent pain (“shooting pain,” “stabbing pain,” “sharp pain,” “splitting pain,” “electric-shock pain,” and “piercing”); (4) fourth model: the independent variable was set to the item of neuropathic pain (“hot-burning pain,” “cold-freezing pain,” “pain caused by light touch,” “itching,” “tingling or pins and needles,” and “numbness”); (5) fifth model: the independent variable was set to the item of continuous pain (“tiring-exhausting,” “sickening,” “fearful,” “punishing-cruel”). The statistical analyses were performed with *R*, ver. 3.6.1.

## 3. Results

### 3.1. Characteristics of the Patients

In Table 1, the characteristics of 23 CLBP patients lasting > 3 months are summarized. The pain site was mostly in the pelvis and buttocks.

### 3.2. Relationship between Muscle Activity and Pain Intensity/Quality with GLMM

Table 2 shows results from GLMM regression modeling for relationship between muscle activity and pain intensity/quality. In the SFMPQ-2 subclass model, neuropathic pain (estimate = -0.62, 95% CI: -1.18 to -0.06) was negatively associated with muscle activity during trunk extension. In the neuropathic pain model, “pain caused by light touch” (estimate = -0.49, 95% CI: -0.94 to -0.05) was negatively associated with muscle activity during trunk extension. On the other hand, in other models (continuous pain model, intermittent pain model, and affective model), all items were not associated with muscle activity during trunk extension.

### 3.3. Relationship between the Distance between the Centroid of Muscle Activity and Pain Site and Pain Intensity/Quality with GLMM

Table 3 shows results from GLMM regression modeling for the relationship between DISTANCE and the pain intensity/quality. In the SFMPQ-2 subclass model, intermittent pain (estimate = 0.53, 95% CI: 0.11 to 0.95) was positively associated with the DISTANCE. In the continuous pain model, “throbbing pain” (estimate = 0.68, 95% CI: 0.13 to 1.23) was positively associated with the DISTANCE. In the intermittent pain model, “splitting pain” (estimate = 0.65, 95% CI: 0.25 to 1.03) was positively associated with the DISTANCE. In the neuropathic pain model, “pain caused by light touch” (estimate = 0.58, 95% CI: 0.18 to 0.99) was positively associated with the DISTANCE. In the affective model, “punishing-cruel” (estimate = 0.61, 95% CI: 0.19 to 1.02) was positively associated with the DISTANCE.

## 4. Discussion

We used GLMM analysis to investigate the relationships among pain site, pain intensity/quality, and muscle activity during trunk extension in 23 patients with CLBP. Regarding pain quality, the results demonstrated that the overall erector spinae muscle activity tended to decrease with an increase in “neuropathic pain” and “pain caused by light touch.” Also, the distance between the centroid of muscle activity and pain site tended to become longer with greater “intermittent pain,” “throbbing pain,” “splitting pain,” “pain caused by light touch,” and “punishing-cruel.” Therefore, the present study showed that the response to these muscle activities varied with the pain quality.

The present study is the first to demonstrate how pain intensity and pain sites are comprehensively associated with muscle activity and muscle activity distribution. In a previous study, erector spinae muscle activity during the extension phase was negatively correlated with pain intensity in the standing, trunk flexion, and re-extension tasks [17]. Similar results were shown in the present study. The altered muscle activity distribution was also reported in CLBP patients in an earlier study [9]. However, a study has also reported that the muscle activity distribution did not systematically change depending on the pain site [18]. Thus, the comprehensive relationship between muscle activity distribution, pain intensity, and pain sites remained controversial. Our present study found that greater pain intensity decreased the overall erector spinae muscle activity during the trunk extension and the distance between the centroid of muscle activity distribution and the pain site became longer. The centroid of muscle activity distribution indicates that near the centroid, there is higher muscle activity. These results indicated that LBP patients inhibited their muscle activity around the pain site during trunk extension. This phenomenon could be explained by the pain adaptation model [2], suggesting that greater pain intensity altered the agonist muscle activity in the painful area. Interestingly, the presence or absence of this phenomenon depended on the pain quality.

Another feature of this study was to investigate the relationships among the pain quality, muscle activity, and muscle activity distribution. The results of the present study showed that “neuropathic pain” and “pain caused by light touch” tended to decrease the overall erector spinae muscle activity. “Pain caused by light touch” indicates superficial pain observed in LBP patients [11]. Our study showed that superficial pain quality could be associated with inhibited overall erector spinae muscle activity. The greater the intensity of “intermittent pain,” “throbbing pain,” “splitting pain,” “pain caused by light touch,” and “punishing-cruel,” the longer the distance between the centroid of muscle activity and pain site tended to be. These results indicated that specific pain quality inhibited muscle activity near the pain site during the trunk extension. “Intermittent pain” indicates the periodic activation of primary nociceptors in response to mechanical stimuli, as mediated by A*δ*-fiber transmission [19]. In other words, “intermittent pain” may reflect the pain quality derived from nociceptive pain. Focusing on the pain quality in relation to LBP-related tissues, “throbbing pain” has been considered muscular pain [20]. In addition, “throbbing pain” has been reported in lumbar facet joint pain [21]. “Splitting pain” has not been examined as a pain quality in LBP-related tissues, but it is thought to be similar to “throbbing pain” and may be characteristic of muscle pain like the latter [22]. “Punishing-cruel” is a pain quality that is classified as emotional, so it is assumed that it does not respond to specific tissues, but it has been reported to be found in fascia pain and muscular pain [20]. Hence, superficial pain, muscular pain, lumbar facet joint pain, and psychological factors may inhibit the muscle activity around the pain site during trunk extension. The present study suggested the possibility of extracting characteristics of pain quality that cause motor adaptation in CLBP patients.

**Figure 3:**
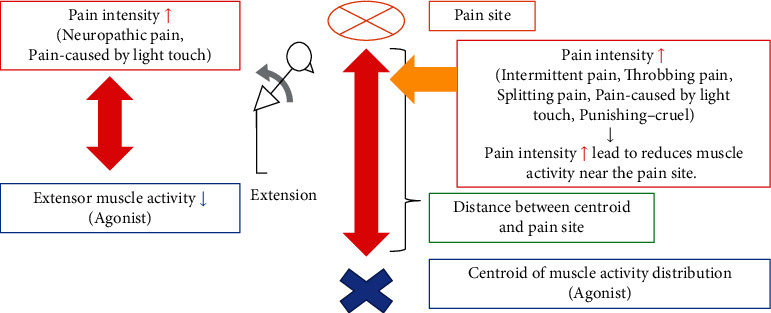
Explanatory model of interaction between pain intensity, pain site, and muscle activity.

Our findings suggest that differences in pain quality can lead to motor adaptation, which reduces agonist muscle activity and further reduces muscle activity near the pain site (Figure 3). Focusing on the treatment, the response of muscle activity varies depending on the pain quality, suggesting the importance of interventions for improving pain intensity by considering the pain quality. In recent years, a variety of interventions have been used for various pain disorders based on the pathogenesis and pain quality [23–25]. For proper muscle activity redistribution, a motor control approach may be necessary, with a combination of interventions appropriate to pain quality.

Our findings suggest the existence of a motor adaptation that suppresses muscle activity near the painful area as the pain intensity increases. Furthermore, the presence or absence of this motor adaptation depended on the pain quality.

This study had several limitations. (1) Patients' lumbar bending and returning tasks were performed based on a previous study; however, although the time was fixed in each movement phase, the speed of patient's movements could not be finely controlled. Therefore, individual differences in velocity can have an impact on muscle activity. (2) The method of listening to the pain site might affect the results. In this study, participants were asked to indicate the most painful area if the pain area was extensive. An extensive pain area rather than the pain site might affect the results. (3) Results were all assessed using pain quality questionnaires, potentially resulting in subjective bias. (4) A variety of LBPs could affect muscle activity values. A variation of the patient LBP sites was observed, and some with and without LBPs occurred in an EMG electrode application site. This might prevent us from extracting the fine muscle activity changes in the cranial to caudal side of the EMG sheet caused by pain. (5) This study included both middle-aged and elderly patients. A previous study reported that muscle activity varies in elderly adults compared to the middle-aged [26]. The age difference might affect muscle activity.

## 5. Conclusion

To our knowledge, this is the first study to investigate the comprehensive associations among pain site, pain intensity/quality, and muscle activity in CLBP patients. Our findings suggest the existence of a motor adaptation that suppresses muscle activity near the painful area as the pain intensity increases. Furthermore, the present study indicates that the presence or absence of this motor adaptation depended on the pain quality. Our findings could help improve motor control in patients with CLBP.

## Figures and Tables

**Table 1:** Characteristics of the patients.

Variables	Mean ± SD
Age in years	71.7 ± 9.3
Gender	Male: 10, female: 13
Pain duration (months)	28.3 ± 44.7
Pain site (cm)	15.0 ± 7.3
RMS (extension phase)	1.01 ± 0.07
Centroid of muscle activity (extension phase) (cm)	4.8 ± 0.2
Distance between the centroid and pain site (cm)	10.2 ± 7.2
NRS	4.7 ± 2.1
SFMPQ-2 total score	30.3 ± 23.8
Continuous pain	12.3 ± 12.0
Intermittent pain	6.6 ± 7.2
Neuropathic pain	5.9 ± 5.8
Affective	5.5 ± 6.2
Throbbing pain	1.8 ± 2.5
Shooting pain	1.8 ± 2.6
Stabbing pain	0.6 ± 1.1
Sharp pain	1.7 ± 2.5
Cramping pain	2.1 ± 3.0
Gnawing pain	0.7 ± 1.5
Hot-burning pain	0.2 ± 0.6
Aching pain	1.8 ± 2.5
Heavy pain	3.4 ± 2.9
Tenderness	1.6 ± 2.9
Splitting pain	0.3 ± 1.0
Electric-shock pain	1.9 ± 2.9
Cold-freezing pain	0.8 ± 1.6
Piercing	0.3 ± 0.8
Pain caused by light touch	0.5 ± 1.1
Itching	0.6 ± 2.0
Tingling or pins and needles	0.8 ± 1.3
Numbness	2.6 ± 2.6

The pain site is indicated as the numerical distance between the top of the electromyography (EMG) sheet and the painful site, with the top of the attached EMG sheet as the starting position (0 cm). RMS: root mean square; NRS: numerical rating scale; SFMPQ-2 : Short-Form McGill Pain Questionnaire-2.

**Table 2:** GLMM regression results for relationship between muscle activity and pain intensity/quality.

Model	Variables	Estimate	Lower CI	Upper CI	Rhat
SFMPQ-2 subclass	Continuous pain	0.35	−0.34	1.04	1.00
	Intermittent pain	−0.12	−0.62	0.38	1.00
	Neuropathic pain	−0.62	−1.18	−0.06	1.00
	Affective	−0.41	−0.92	0.10	1.00

Continuous pain	Throbbing pain	−0.03	−0.80	0.75	1.00
	Cramping pain	0.29	−0.73	1.29	1.00
	Gnawing pain	−0.17	−0.74	0.41	1.00
	Aching pain	−0.13	−1.06	0.81	1.00
	Heavy pain	0.10	−0.72	0.92	1.00
	Tenderness	−0.49	−1.45	0.48	1.00

Intermittent pain	Shooting pain	−0.05	−0.75	0.65	1.00
	Stabbing pain	−0.06	−0.69	0.56	1.00
	Sharp pain	0.21	−0.41	0.83	1.00
	Splitting pain	−0.29	−1.07	0.48	1.00
	Electric-shock pain	−0.09	−0.66	0.48	1.00
	Piercing	−0.08	−0.73	0.58	1.00

Neuropathic pain	Hot-burning pain	0.33	−0.22	0.86	1.00
	Cold-freezing pain	−0.25	−0.71	0.21	1.00
	Pain caused by light touch	−0.49	−0.94	−0.05	1.00
	Itching	−0.02	−0.50	0.49	1.00
	Tingling or pins and needles	0.02	−0.54	0.60	1.00
	Numbness	−0.23	−0.74	0.30	1.00

Affective	Tiring-exhausting	0.07	−0.60	0.74	1.00
	Sickening	−0.32	−1.04	0.39	1.00
	Fearful	0.04	−0.65	0.73	1.00
	Punishing-cruel	−0.16	−0.82	0.49	1.00

CI: credible intervals.

**Table 3:** GLMM regression results for relationship between the distance between the centroid of muscle activity and pain site and pain intensity/quality.

Model	Variables	Estimate	Lower CI	Upper CI	Rhat
SFMPQ-2 subclass	Continuous pain	0.46	−0.13	1.03	1.00
	Intermittent pain	0.53	0.11	0.95	1.00
	Neuropathic pain	−0.07	−0.53	0.40	1.00
	Affective	−0.29	−0.72	0.14	1.00

Continuous pain	Throbbing pain	0.68	0.13	1.23	1.00
	Cramping pain	0.14	−0.57	0.85	1.00
	Gnawing pain	0.09	−0.32	0.50	1.00
	Aching pain	−0.01	−0.67	0.66	1.00
	Heavy pain	−0.13	−0.71	0.45	1.00
	Tenderness	0.07	−0.61	0.75	1.00

Intermittent pain	Shooting pain	0.10	−0.25	0.45	1.00
	Stabbing pain	0.08	−0.23	0.40	1.00
	Sharp pain	0.19	−0.12	0.50	1.00
	Splitting pain	0.65	0.25	1.03	1.00
	Electric-shock pain	−0.08	−0.36	0.21	1.00
	Piercing	0.08	−0.25	0.40	1.00

Neuropathic pain	Hot-burning pain	0.32	−0.19	0.81	1.00
	Cold-freezing pain	0.09	−0.33	0.53	1.00
	Pain caused by light touch	0.58	0.18	0.99	1.00
	Itching	0.06	−0.38	0.50	1.00
	Tingling or pins and needles	−0.02	−0.53	0.51	1.00
	Numbness	0.04	−0.44	0.51	1.00

Affective	Tiring-exhausting	−0.39	−0.82	0.03	1.00
	Sickening	0.13	−0.32	0.58	1.00
	Fearful	0.19	−0.26	0.63	1.00
	Punishing-cruel	0.61	0.19	1.02	1.00

CI: credible intervals.

## Data Availability

All data generated or analyzed during this study are included within this article.
